# Thermal buckling and postbuckling of functionally graded multilayer GPL-reinforced composite beams on nonlinear elastic foundations

**DOI:** 10.1016/j.heliyon.2023.e19549

**Published:** 2023-08-28

**Authors:** Ying Lv, Jing Zhang, Lianhe Li

**Affiliations:** a Mathematics Science College, Inner Mongolia Normal University, Hohhot, 010022, China; b Center for Applied Mathematics Inner Mongolia, Hohhot, 010022, China; c Key Laboratory of Infinite-dimensional Hamiltonian System and Its Algorithm Application, Ministry of Education, Hohhot, 010022, China

**Keywords:** Differential quadrature method, Functionally graded materials, Thermal buckling, Thermal postbuckling, Graphene platelet

## Abstract

Under the influence of axial forces and uniform temperature variations, the thermal buckling and postbuckling of composite beams reinforced of functionally graded multilayer graphene platelets (GPLs) resting on nonlinear elastic foundations are examined. The Halpin-Tsai model is used to calculate the elastic modulus of each layer of GPL-reinforced composite (GPLRC). According to the virtual work principle, the nonlinear governing equations for the beam are obtained from the first-order shear deformation beam theory. The impact of axial force and nonlinear elastic foundation on thermal buckling and postbuckling is discussed using the differential quadrature method (DQM), and the analytical expression is given by the two-step perturbation method (TSPM). The effects of axial force, boundary conditions, slenderness ratio, GPL geometry, GPL weight fraction, GPL distribution pattern, and elastic foundation coefficient on thermal buckling and postbuckling are examined through parameter analysis.

## 1 Introduction

With the development of science and technology, new energy, aerospace and other industries have higher and higher requirements for material properties. Functionally graded materials (FGM) were launched for the first time in 1987 [1], which is a new composite materials whose properties can be continuously changed [2]. FGMs improve the connection strength while also lowering thermal and residual stress at the interface. These advantages greatly facilitate people to meet the needs of different properties like high-temperature and low-temperature resistance in structural design, making the structure more reliable and integrated.

Graphene, a hexagonal 2-dimensional material based on carbon atoms, was discovered by Geim et al. [3] and Novoselov et al. [4]. Lee et al. [5] found that it has strong elastic properties and tensile strength, which are much higher than traditional materials. It was also found that graphene is the hardest material measured, with tensile strength of about 130 GPa, more than 100 times that of the best steel in nature [6]. Graphene has good performance in thermal, dynamic and electrical aspects [[7], [8], [9]], it becomes an ideal reinforcing material when combined with other materials [[10], [11], [12]]. The effect of GPL/epoxy nanocomposites at different GPL sizes was studied by Wang et al. [13], who demonstrated that larger GPL sizes should reduce the strength of the composites and increase the elastic modulus.

In 2017, Yang et al. [14] and Feng et al. [15] first presented the notion of functionally graded in graphene materials for the first time and obtained a novel graphene platelet-reinforced functionally graded material. They found that when graphene is distributed in the matrix material according to a specific distribution, it can be further improved the strength, stiffness, and critical buckling load of the composite. Using DQM, Wu et al. [16,17] investigated the dynamic instability of GPLRC beams as well as the buckling and postbuckling of plates in the thermal environment. Song et al. [18] explored cracked GPLRC beams on foundations for thermal buckling and postbuckling. The stability under various conditions was described by Wang et al. [19] as they examined the buckling and postbuckling of GPLRC plates in electric fields. Using TSPM, Song et al. [20] explored the biaxial pressure buckling and postbuckling behavior of functionally graded GPLRC plates at lower graphene reinforcement contents. Shen et al. [21] studied the mechanical performance of functionally graded GPLRC beams in postbuckling and nonlinear bending under thermal environments.

In addition, other methods had been used to analyze the mechanical performance of beams, plates, shells. In-plane bi-directional functionally graded plates and multidirectional functionally graded plates were subjected to free vibration and bending analyses by Lieu et al. [[22], [23], [24]] using isogeometric analysis (IGA). The free vibration and buckling of functionally graded GPLRC porous beams were examined by Kitipornchai et al. [25] using the Ritz method. Feng et al. [26] investigated the nonlinear free vibration of graphene platelet-reinforced multilayer polymer composite beams with nonuniform distribution in the thickness region, and obtained the impact of GPL and size on the intrinsic frequency. A rotating stiffened toroidal functionally graded GPLRC shell segment was obtained by Nguyen et al. [27] to give free vibration in a temperature environment. The nonlinear eigenvalue buckling of a functionally graded multilayer GPLRC cylindrical shell was explored by Wang et al. [28] using the finite element method. Zhao et al. [29] showed the bending and vibration behavior of functionally graded multilayer GPLRC trapezoidal plates and found that the static and dynamic deflection of the plates decreased as either of the two bottom angles became smaller. The Longe-Kuta approach is used in the work of [30] to provide the vibration and nonlinear dynamic response of GPLRC plates on a viscoelastic Pasternak medium. The nonlinear vibration of functionally graded GPLRC cylindrical plates under transverse excitation was studied by Niu et al. [31] using the Navier method. To examine the impact of microstructure and length scale on the buckling and bending of functionally graded multilayer GPLRC plates, Ghandourah et al. [32] combined the continuous nonlocal strain graded theory with the quasi-3D hyperbolic higher order shear deformation plate theory. Daikh et al. [33] first proposed the static bending response and stress distribution of functionally graded multilayer sandwich nanoplates under the variable Winkler foundation model. In the works of [34,35], the functionally graded GPLRC plates and the temperature-dependent functionally graded carbon nanotube-reinforced composite laminated double curved shallow shell were employed to calculate the nonlinear dynamic response and vibration problems. Ref. [36] studied the free vibration and buckling of functionally graded multilayer GPLRC perforated plates, which proposed a meshless radial point interpolation approach. Galerkin method and the multiple timescale method were utilized in Reference [37] to examine the nonlinear vibration of functionally graded multilayer GPLRC beams. The connection between nonlinear buckling and postbuckling was demonstrated by Ramezani et al. [38] predicated on the higher-order shear deformation theory.

As far as the authors’ knowledge, the buckling and postbuckling of GPLRC beams have not been studied on the basis of nonlinear elastic foundation, thermal environment and axial force. Therefore, this paper focuses on buckling and postbuckling of functionally graded multilayer GPLRC beams that are situated on a nonlinear elastic foundation in a thermal environment as a result of the combined influence of uniform temperature change and axial forces. The first-order shear deformation beam theory is the theoretical foundation from which the governing equation of the beam is derived. DQM is used to determine the critical buckling temperature and the postbuckling temperature-deflection path of the beam. We also use TSPM to calculate thermal buckling and postbuckling. The effects of the elastic foundation coefficient, boundary conditions, slenderness ratio, GPL distribution mode, GPL geometry, GPL weight percentage, and the axial force are investigated by parameter analysis.

## 2 The effective properties of GPLRC

### 2.1 Problem description

**Fig. 1:**
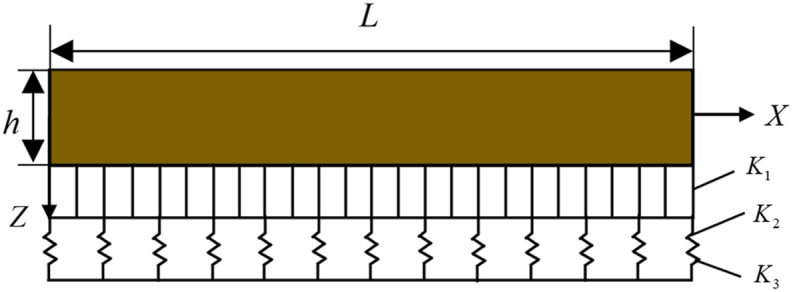
Configuration diagram of a GPLRC beam with functionally graded on nonlinear elastic foundation.

The model of GPLs functionally graded multilayer beams is illustrated in Fig. 1, with coordinates X and Z pointing in the direction of the length L and thickness h of the beam separately. By bonding, the graphene in each layer of the polymer matrix material is uniformly distributed, but the graphene weight fraction in different layers is different. $K_{1},K_{2},K_{3}$ are the linear Winkler stiffness, shearing layer stiffness, and nonlinear Winkler stiffness of the foundation, respectively.

### 2.2 Material parameters

**Fig. 2:**
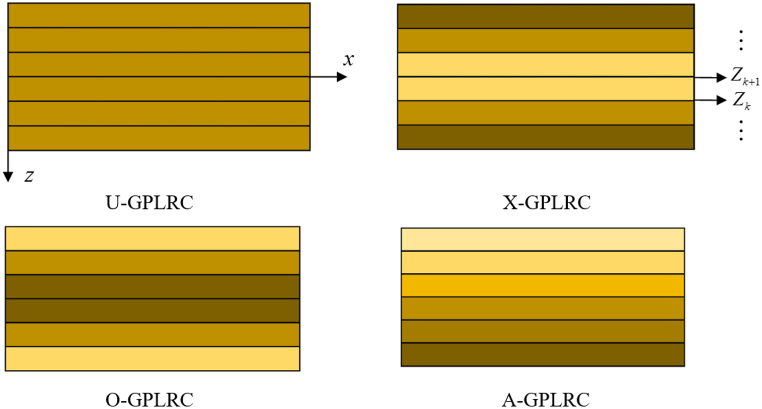
GPL distribution model.

The four distribution modes of GPL nanofillers are shown on Fig. 2. Among them, U-GPLRC indicates that the GPL concentration is uniformly distributed in each layer, near the top and bottom layers, X-GPLRC contains more GPL, whereas intermediate concentration is lower, O-GPLRC has the reversed pattern of GPL content from X-GPLRC, i.e., lower GPL content near the top and bottom layers and higher intermediate concentration, and A-GPLRC has gradually higher GPL content from the top to the bottom layers, and it is easy to see that all the other three distribution patterns have symmetry except A- GPLRC.

For various distribution patterns, the GPL volume fraction for the *k* th ($k=1,2,3,...,N_{L}$) layer is given by [14]

(1) $$U-\text{GPLRC}:V_{\text{GPL}}\left(k\right)=V_{\text{GPL}}^{*}\text{,}$$

(2) $$X-\text{GPLRC}:V_{\text{GPL}}\left(k\right)=\frac{2V_{\text{GPL}}^{*}\left|2k-N_{L}-1\right|}{N_{L}}\text{,}$$

(3) $$O-\text{GPLRC}:V_{\text{GPL}}\left(k\right)=2V_{\text{GPL}}^{*}\left(1-\frac{\left|2k-N_{L}-1\right|}{N_{L}}\right)\text{,}$$

(4) $$A-\text{GPLRC}:V_{\text{GPL}}\left(k\right)=\frac{V_{\text{GPL}}^{*}\left(2k-1\right)}{N_{L}}$$

The total number of layers in the beam is $N_{L}$, and $V_{\text{GPL}}^{*}$ represents for the total volume fraction, the link between $V_{\text{GPL}}^{*}$ and the GPL weight fraction $W_{\text{GPL}}$ is given by [39]

(5) $$V_{\text{GPL}}^{*}=\frac{W_{\text{GPL}}}{W_{\text{GPL}}+\left(\rho_{\text{GPL}}/\rho_{m}\right)\left(1-W_{\text{GPL}}\right)}$$

where the polymer matrix and GPL's mass density are $\rho_{m}$ and $\rho_{\text{GPL}}$, respectively.

The Halpin-Tsai micromechanical model predicts the modulus of elasticity E [[40], [41], [42]]:

(6) $$E=\frac{3}{8}\frac{1+\xi_{L}\eta_{L}V_{\text{GPL}}}{1-\eta_{L}V_{\text{GPL}}}\times E_{m}+\frac{5}{8}\frac{1+\xi_{T}\eta_{T}V_{\text{GPL}}}{1-\eta_{T}V_{\text{GPL}}}\times E_{m}$$

where $\eta_{L}$ and $\eta_{T}$ are depicted by

(7) $$\eta_{L}=\frac{\frac{E_{\text{GPL}}}{E_{m}}-1}{\frac{E_{\text{GPL}}}{E_{m}}+\xi_{L}},\eta_{T}=\frac{\frac{E_{\text{GPL}}}{E_{m}}-1}{\frac{E_{\text{GPL}}}{E_{m}}+\xi_{T}}$$

Where $E_{m}$, $E_{\text{GPL}}$ stand for the elastic modulus of the polymer matrix and GPL, and $\xi_{L}$, $\xi_{T}$ stand for the filling geometry factor of GPL [41]

(8) $$\xi_{L}=2\frac{a_{\text{GPL}}}{t_{\text{GPL}}},\xi_{T}=2\frac{b_{\text{GPL}}}{t_{\text{GPL}}}$$

where $a_{\text{GPL}},t_{\text{GPL}},b_{\text{GPL}}$ represent the length, thickness, width of GPLs, respectively. $\xi_{L}$ can represent

(9) $$\xi_{L}=2\frac{a_{\text{GPL}}}{b_{\text{GPL}}}\times \frac{b_{\text{GPL}}}{t_{\text{GPL}}}$$

The elastic modulus of each layer under different distribution patterns can be obtained by putting Eqs. (1), (2), (3), (4), (5) and Eqs. (7), (8), (9) into Eq. (6). According to the mixing rule [43], Poisson's ratio v, coefficient of thermal expansion α can be determined

(10) $$v=v_{m}V_{m}+v_{\text{GPL}}V_{\text{GPL}}$$

(11) $$\alpha =\alpha_{m}V_{m}+\alpha_{\text{GPL}}V_{\text{GPL}}$$

where $\alpha_{m},\alpha_{\text{GPL}}$ refer to the thermal expansion coefficient of the polymer matrix, GPL, and the Poisson's ratio of GPL to matrix is $v_{\text{GPL}}$ to $v_{m}$, with the volume fraction satisfying the relation $V_{m}=1-V_{\text{GPL}}$.

## 3 Equations of motion

Let the beam have no stress under the initial temperature $T_{0}$ and be exposed to axial forces and homogeneous temperature changes $\Delta T=T-T_{0}$. The first-order shear deformation beam theory yields the displacement field for the beam [44]

(12) $$\left\{ \begin{array}{l} \bar{U}\left(X,Z\right)=U\left(X\right)+Z\psi \left(X\right)\\ \bar{W}\left(X,Z\right)=W\left(X\right) \end{array}\right.$$

where ψ is the rotation of the beam cross section, U and W are the displacement components in the mid-plane of the beam, as well as the displacements $\bar{U}$ and $\bar{W}$ are along the *X*-axis and *Z*-axis of the beam, respectively. Consider the von Kármán type nonlinear strain-displacement relationship, Eq. (12) can be obtained

(13) $$\left\{ \begin{array}{l} \varepsilon_{XX}=\frac{\partial U}{\partial X}+\frac{1}{2}{\left(\frac{\partial W}{\partial X}\right)}^{2}+Z\frac{\partial \psi }{\partial X}\\ \gamma_{XZ}=\frac{\partial W}{\partial X}+\psi \end{array}\right.$$

The linear stress-strain relationship is obtained according to Eq. (13):

(14) $$\left\{ \begin{array}{l} \sigma_{XX}=Q_{11}\left[\frac{\partial U}{\partial X}+\frac{1}{2}{\left(\frac{\partial W}{\partial X}\right)}^{2}+Z\frac{\partial \psi }{\partial X}-\alpha \Delta T\right]\\ \tau_{XZ}=Q_{55}\left(\frac{\partial W}{\partial X}+\psi \right) \end{array}\right.$$

according to Eqs. (6), (10), the reduced stiffness is given by:

(15) $$Q_{11}=\frac{E}{1-v^{2}},Q_{55}=\frac{E}{2\left(1+v\right)}$$

The control equation for the beam is derived using the principle of virtual displacement

(16) $$\delta \gamma_{P}-\delta V=0$$

Where, the imaginary work $\delta \gamma_{P}\;$ and imaginary strain energy δV are given by

(17) $$\delta \gamma_{P}=\int_{0}^{L}\bar{P}\frac{\partial W}{\partial X}\frac{\partial \delta W}{\partial X}dX$$

(18) $$\delta V=\int_{0}^{L}\int_{-h/2}^{h/2}\left(\sigma_{XX}\varepsilon_{XX}+\tau_{XZ}\gamma_{XZ}\right)\;dZ\;dX+\int_{0}^{L}\left(K_{1}W\delta W+K_{2}\frac{\partial W}{\partial X}\frac{\partial \delta W}{\partial X}+K_{3}W^{3}\delta W\right)\;dX$$

Substituting $\delta \gamma_{P}\;$ and δV from Eqs. (17-18) into Eq. (16)

(19) $$\begin{array}{l} 0=\int_{0}^{L}\{\frac{\partial N_{X}}{\partial X}\delta U+\left(\frac{\partial M_{X}}{\partial X}-Q_{X}\right)\delta \psi +\left[\frac{\partial Q_{X}}{\partial X}-K_{1}W+K_{2}\frac{\partial^{2}W}{\partial X^{2}}-K_{3}W^{3}+\left(N_{X}-\bar{P}\right)\frac{\partial^{2}W}{\partial X^{2}}\right]\delta W\}\text{dX}\\ {-\left(N_{X}\delta U\right)|}_{0}^{L}{-\left(M_{X}\delta \psi \right)|}_{0}^{L}{-\left[\left(N_{X}\frac{\partial W}{\partial X}+Q_{X}+K_{2}\frac{\partial W}{\partial X}-\bar{P}‾\frac{\partial W}{\partial X}\right)\delta W\right]|}_{0}^{L} \end{array}$$

where $N_{X}$ is the normal force, $M_{X}$ is the bending moment, and $Q_{X}$ is the transverse shear force. Eq. (14) is substituted into the following equation

(20) $$N_{X}=\int_{-h/2}^{h/2}\sigma_{XX}\text{dZ}=A_{11}\frac{\partial U}{\partial X}+B_{11}\frac{\partial \psi }{\partial X}+\frac{1}{2}A_{11}{\left(\frac{\partial W}{\partial X}\right)}^{2}-N_{X}^{T}$$

(21) $$M_{X}=\int_{-h/2}^{h/2}\sigma_{XX}Z\;\text{dZ}=B_{11}\frac{\partial U}{\partial X}+D_{11}\frac{\partial \psi }{\partial X}+\frac{1}{2}B_{11}{\left(\frac{\partial W}{\partial X}\right)}^{2}-M_{X}^{T}$$

(22) $$Q_{X}=\int_{-h/2}^{h/2}\tau_{XZ}\;\text{dZ}=\kappa A_{55}\left(\frac{\partial W}{\partial X}+\psi \right)$$

where the shear correction coefficient κ=5/6 [44]. Substitute Eq. (11) into thermally induced forces and moments:

(23) $$\left\{N_{X}^{T},M_{X}^{T}\right\}=\int_{-h/2}^{h/2}Q_{11}\alpha \;\Delta T\left\{1,Z\right\}\text{dZ}=\sum_{k=1}^{N_{L}}\int_{Z_{k}}^{Z_{k+1}}Q_{11}^{\left(k\right)}\alpha^{\left(k\right)}\;\Delta T\left\{1,Z\right\}\text{dZ}$$

Along the thickness direction, has $Z_{k}$ and $Z_{k+1}$ as the lower and upper surface heights of the k th layer beam, respectively. The stiffness component is defined as:

(24) $$\left\{A_{11},B_{11},D_{11}\right\}=\int_{-h/2}^{h/2}Q_{11}\left\{1,Z,Z^{2}\right\}\text{dZ}=\sum_{k=1}^{N_{L}}\int_{Z_{k}}^{Z_{k+1}}Q_{11}^{\left(k\right)}\left\{1,Z,Z^{2}\right\}\text{dZ}$$

(25) $$A_{55}=\int_{-h/2}^{h/2}Q_{55}\;\text{dZ}=\sum_{k=1}^{N_{L}}\int_{z_{k}}^{z_{k+1}}Q_{55}^{\left(k\right)}\text{dZ}$$

The governing equations are found by setting the δU,δW and δψ coefficients in Eq. (19) to zero:

(26) $$\frac{\partial N_{X}}{\partial X}=0$$

(27) $$\frac{\partial Q_{X}}{\partial X}-K_{1}W+K_{2}\frac{\partial^{2}W}{\partial X^{2}}-K_{3}W^{3}+\left(N_{X}-\bar{P}\right)\frac{\partial^{2}W}{\partial X^{2}}=0$$

(28) $$\frac{\partial M_{X}}{\partial X}-Q_{X}=0$$

Consider the following boundary conditions for the beam that is both clamped and hinged:

Clamped (C):

(29) U=0,W=0,ψ=0

Hinged (H):

(30) $$U=0,W=0,M_{X}=0$$

Substituting Eq. (15) into Eqs. (23), (24), (25) and Eqs. (20), (21), (22), the control equations and related boundary conditions are obtained according to Eqs. (26), (27), (28) and Eqs. (29), (30)

(31) $$A_{11}\frac{\partial^{2}U}{\partial X^{2}}+B_{11}\frac{\partial^{2}\psi }{\partial X^{2}}+A_{11}\frac{\partial W}{\partial X}\frac{\partial^{2}W}{\partial X^{2}}-\frac{\partial N_{X}^{T}}{\partial X}=0\text{,}$$

(32) $$\kappa A_{55}\left(\frac{\partial^{2}W}{\partial X^{2}}+\frac{\partial \psi }{\partial X}\right)-K_{1}W+K_{2}\frac{\partial^{2}W}{\partial X^{2}}-K_{3}W^{3}+\left[A_{11}\frac{\partial U}{\partial X}+B_{11}\frac{\partial \psi }{\partial X}+\frac{1}{2}A_{11}{\left(\frac{\partial W}{\partial X}\right)}^{2}-N_{X}^{T}-\bar{P}\right]\frac{\partial^{2}W}{\partial X^{2}}=0$$

(33) $$B_{11}\frac{\partial^{2}U}{\partial X^{2}}+D_{11}\frac{\partial^{2}\psi }{\partial X^{2}}+B_{11}\frac{\partial W}{\partial X}\frac{\partial^{2}W}{\partial X^{2}}-\frac{\partial M_{X}^{T}}{\partial X}-\kappa A_{55}\left(\frac{\partial W}{\partial X}+\psi \right)=0$$

(34) U=0,W=0,ψ=0

(35) $$U=0,W=0,B_{11}\frac{\partial U}{\partial X}+D_{11}\frac{\partial \psi }{\partial X}+\frac{1}{2}B_{11}{\left(\frac{\partial W}{\partial X}\right)}^{2}-M_{X}^{T}=0$$

## 4 Solution procedure

### 4.1 Introduce dimensionless quantities

(36) $$\begin{array}{l} x=X/L,\eta =h/L,\left\{u,w\right\}=\left\{U,W\right\}/h,\varphi =\psi ,\left\{P,P^{T},M^{T}\right\}=\left\{\bar{P},N_{x}^{T},M_{x}^{T}/h\right\}/A_{110}\text{,}\\ \left\{k_{1},k_{2},k_{3}\right\}=\left\{K_{1}L^{2},K_{2},L^{2}h^{2}K_{3}\right\}/A_{110},\left\{a_{11},a_{55},b_{11},d_{11}\right\}=\left\{A_{11},\kappa A_{55},B_{11}/h,D_{11}/h^{2}\right\}/A_{110} \end{array}$$

where $A_{110}$ represents the $A_{11}$ value of the pure matrix material. Through equation (36), the control equations (31), (32), (33) and boundary conditions (34–35) are converted to dimensionless control equations and dimensionless boundary conditions

(37) $$a_{11}\frac{\partial^{2}u}{\partial x^{2}}+b_{11}\frac{\partial^{2}\varphi }{\partial x^{2}}+a_{11}\eta \frac{\partial w}{\partial x}\frac{\partial^{2}w}{\partial x^{2}}=0$$

(38) $$a_{55}\left(\frac{\partial^{2}w}{\partial x^{2}}+\frac{1}{\eta }\frac{\partial \varphi }{\partial x}\right)+\left[a_{11}\eta \frac{\partial u}{\partial x}+b_{11}\eta \frac{\partial \varphi }{\partial x}+\frac{1}{2}a_{11}\eta^{2}{\left(\frac{\partial w}{\partial x}\right)}^{2}-P^{T}-P\right]\frac{\partial^{2}w}{\partial x^{2}}-k_{1}w+k_{2}\frac{\partial^{2}w}{\partial x^{2}}-k_{1}w^{3}=0$$

(39) $$b_{11}\frac{\partial^{2}u}{\partial x^{2}}+d_{11}\frac{\partial^{2}\varphi }{\partial x^{2}}+b_{11}\eta \frac{\partial w}{\partial x}\frac{\partial^{2}w}{\partial x^{2}}-\frac{a_{55}}{\eta }\left(\frac{\partial w}{\partial x}+\frac{1}{\eta }\varphi \right)=0$$

(40) u=0,w=0,φ=0

(41) $$u=0,w=0,b_{11}\frac{\partial u}{\partial x}+d_{11}\frac{\partial \varphi }{\partial x}+\frac{1}{2}b_{11}\eta {\left(\frac{\partial w}{\partial x}\right)}^{2}-\frac{1}{\eta }M^{T}=0$$

For the numerical method of differential equations, DQM is simpler and faster than the analytical method. However, DQM can only solve problems in regular regions, and no specific expressions can be obtained. According to the DQM [[45], [46], [47]], the j th partial derivative from the displacement variables u,w,φ to ζ as a linear weighted sum of $u_{m},w_{m},\varphi_{m}$

(42) $$\begin{array}{l} \left\{u,w,\varphi \right\}=\sum_{m=1}^{N}l_{m}\left(x\right)\left\{u_{m},w_{m},\varphi_{m}\right\}\text{,}\\ {\frac{\partial^{j}}{\partial x^{j}}\left\{u,w,\varphi \right\}|}_{x=x_{i}}=\sum_{m=1}^{N}C_{m}^{\left(j\right)}\left\{u_{m},w_{m},\varphi_{m}\right\} \end{array}$$

where $\left\{u_{m},w_{m},\varphi_{m}\right\}$ is {u,w,φ} at $x=x_{m}$; $l_{m}\left(x\right)$ is a polynomial of Lagrange interpolation; $C_{m}^{\left(j\right)}$ is the j th partial derivative of the displacement component relative to x. The recurrence formula is given in Refs. [48,49], and the total number of grid points on [0,1] is *N*. The Chebyshev formula is used to discretize the axes of the beam:

(43) $$x_{i}=\frac{1}{2}\left[1-\cos\frac{\pi \left(i-1\right)}{N-1}\right],i=1,2,\cdots ,N$$

Substituting Eqs. (42), (43) into the dimensionless control Eqs. (37), (38), (39) and dimensionless boundary conditions (40–41), the discretized control equations and boundary conditions are obtained:

(44) $$a_{11}\sum_{m=1}^{N}C_{im}^{\left(2\right)}u_{m}+b_{11}\sum_{m=1}^{N}C_{im}^{\left(2\right)}\varphi_{m}+a_{11}\eta \sum_{m=1}^{N}C_{im}^{\left(1\right)}w_{m}\sum_{m=1}^{N}C_{im}^{\left(2\right)}w_{m}=0$$

(45) $$\left[a_{11}\eta \sum_{m=1}^{N}C_{im}^{\left(1\right)}u_{m}+b_{11}\eta \sum_{m=1}^{N}C_{im}^{\left(1\right)}\varphi_{m}+\frac{1}{2}a_{11}\eta^{2}{\left(\sum_{m=1}^{N}C_{im}^{\left(1\right)}w_{m}\right)}^{2}-P^{T}-P\right]\times \sum_{m=1}^{N}C_{im}^{\left(2\right)}w_{m}+a_{55}\left(\sum_{m=1}^{N}C_{im}^{\left(2\right)}w_{m}+\frac{1}{\eta }\sum_{m=1}^{N}C_{im}^{\left(1\right)}\varphi_{m}\right)-k_{1}w_{i}+k_{2}\sum_{m=1}^{N}C_{im}^{\left(2\right)}w_{m}-k_{3}w_{i}^{3}=0$$

(46) $$b_{11}\sum_{m=1}^{N}C_{im}^{\left(2\right)}u_{m}+d_{11}\sum_{m=1}^{N}C_{im}^{\left(2\right)}\varphi_{m}+b_{11}\eta \sum_{m=1}^{N}C_{im}^{\left(1\right)}w_{m}\sum_{m=1}^{N}C_{im}^{\left(2\right)}w_{m}-\frac{a_{55}}{\eta }\left(\sum_{m=1}^{N}C_{im}^{\left(1\right)}w_{m}+\frac{1}{\eta }\varphi_{i}\right)=0$$

for clamping ends at x=0,1

(47) $$u_{1}=0,w_{1}=0,\varphi_{1}=0$$

(48) $$u_{N}=0,w_{N}=0,\varphi_{N}=0$$

for hinged ends at x=0,1

(49) $$u_{1}=0,w_{1}=0,b_{11}\sum_{m=1}^{N}C_{1m}^{\left(1\right)}u_{m}+d_{11}\sum_{m=1}^{N}C_{1m}^{\left(1\right)}\varphi_{m}+\frac{1}{2}b_{11}\eta {\left(\sum_{m=1}^{N}C_{1m}^{\left(1\right)}w_{m}\right)}^{2}-{\frac{1}{\eta }M^{T}|}_{x=x_{1}}=0$$

(50) $$u_{N}=0,w_{N}=0,b_{11}\sum_{m=1}^{N}C_{Nm}^{\left(1\right)}u_{m}+d_{11}\sum_{m=1}^{N}C_{Nm}^{\left(1\right)}\varphi_{m}+\frac{1}{2}b_{11}\eta {\left(\sum_{m=1}^{N}C_{Nm}^{\left(1\right)}w_{m}\right)}^{2}-{\frac{1}{\eta }M^{T}|}_{x=x_{N}}=0$$

Replacing the relevant boundary conditions (47–50) with the discretized control Eqs. (44), (45), (46) yields a nonlinear system controlling the buckling and postbuckling behavior of beams on the nonlinear foundation

(52) $$\left\{{Κ}_{L}-\Delta T{Κ}_{T}-P{Κ}_{P}+{Κ}_{\text{NL}1}+{Κ}_{\text{NL}2}\right\}d=0$$

in which the displacement component $d={\left\{{\left\{u_{i}\right\}}^{T},{\left\{w_{i}\right\}}^{T},{\left\{\varphi_{i}\right\}}^{T}\right\}}^{T},i=1,2,\cdots ,N$; ${Κ}_{L}$ is a matrix of constant coefficients; the coefficient matrices ${Κ}_{T}$ and ${Κ}_{P}$, which are connected to the temperature rise ΔT and axial force P, respectively; while the ${Κ}_{NL1}$ and ${Κ}_{NL2}$ components are linear and quadratic functions of d, respectively.

By removing the nonlinear term in Eq. (51), $P_{\text{cr}}$ or $\Delta T_{\text{cr}}$ can be obtained. The postbuckling temperature is obtained according to the iterative algorithm by Liew et al. [50].

## 5 Numerical results and analysis

The length, width, height of the GPLs are $a_{\text{GPL}}=2.5\;\mu m,b_{\text{GPL}}=1.5\;\mu m,t_{\text{GPL}}=1.5\;\text{nm}$, respectively. The total thickness of the beams is *h* = 0.0lm. Assuming that epoxy and GPL material properties are independent of temperature [17], as shown in Table 1.

**Table 1:** Material properties.

	Epoxy [52]	GPL [51]
Young's modulus (GPa)	3.0	1010
Density ($kg\;m^{-3}$)	1200	1062.5
Poisson's ratio	0.34	0.186
Thermal expansion coeffificient ($\times 10^{-6}/K$)	60	5.0

### 5.1 Convergence verification

In order to verify the analytical results of this paper, convergence is first verified and Table 2 compares the results for different amounts of grid points and number of layers. $w_{c}$ is the dimensionless center deflection, $\Delta T_{\text{cr}}$ is the critical buckling temperature rise, ΔT is the postbuckling temperature when $w_{c}=1$, and $P_{\text{cr}}$ is the critical buckling load. When the number of layers $N_{L}=30$ and the grid point N=13, it is seen that the results converge. For convenience of manufacture and cost, in the numerical calculations below N=13 and $N_{L}=10$ are used.

**Table 2:** Results for thermal buckling and postbuckling at different grid points and number of layers. (C–C, X-GPLRC, $L/h=20,W_{\text{GPL}}=0.3\%,\left(k_{1},k_{2},k_{3}\right)=\left(0,0,0\right)$).

$\begin{array}{l} N\\ \left(N_{L}=10\right) \end{array}$	$P_{\text{cr}}$	$\Delta T_{\text{cr}}$	$\begin{array}{l} \Delta T\\ \left(w_{c}=1.0\right) \end{array}$	$\begin{array}{l} N_{L}\\ \left(N=13\right) \end{array}$	$P_{\text{cr}}$	$\Delta T_{\text{cr}}$	$\begin{array}{l} \Delta T\\ \left(w_{c}=1.0\right) \end{array}$
7	0.0196	164.5660	176.4924	6	0.0193	162.1498	265.3198
9	0.0196	164.4333	255.7043	10	0.0196	164.426	267.5998
11	0.0196	164.4259	267.3288	20	0.0197	165.3857	268.5610
13	0.0196	164.4260	267.5998	30	0.0198	165.5633	268.7390
15	0.0196	164.4260	267.6059	40	0.0198	165.6255	268.8013
17	0.0196	164.4260	267.6060	50	0.0198	165.6543	268.8301

The next step is comparison verification. Table 3 compares the results with those of Wu et al. [17] and calculates the critical buckling temperature rise of each boundary condition under different slenderness ratio when $k_{1}=0,k_{2}=0$. In order to verify the accuracy of DQM, according to TSPM proposed by Shen et al. [53]. Table 4, Table 5 present the computed values for the critical buckling loads and postbuckling temperature changes, respectively. The precision is confirmed through a comparison with the findings presented in Refs. [14,18].

**Table 3:** Critical buckling temperature rise with various boundary conditions and slenderness ratios. (X-GPLRC, $W_{\text{GPL}}=0.3\%$).

		L/h=25	L/h=30	L/h=35	L/h=40
C–C	Present	106.6032	74.5575	55.0133	42.2378
	Ref. [17]	106.60	74.557	55.013	42.238
C–H	Present	55.0793	38.4038	28.2838	21.6891
	Ref. [17]	55.079	38.404	28.284	21.689
H–H	Present	27.1218	18.8685	13.8777	10.6326
	Ref. [17]	27.122	18.869	13.878	10.633

**Table 4:** Critical buckling loads for with and without elastic foundation under different distribution modes. (H–H, $L/h=10,W_{\text{GPL}}=0.3\%$).

Source	$\left(k_{1},k_{2}\right)=\left(0,0\right)$				$\left(k_{1},k_{2}\right)=\left(0.1,0.02\right)$			
	X	U	O	A	X	U	O	A
present	0.0196	0.0159	0.0122	0.0147	0.0497	0.0461	0.0423	0.0448
TSPM	0.0196	0.0159	0.0122	0.0147	0.0497	0.0461	0.0423	0.0448
Ref. [14]	0.0196	0.0159	0.0122	0.0147	0.0497	0.0461	0.0423	0.0448

**Table 5:** The thermal postbuckling load-deflection relationship of X-GPLRC beams under C–C boundary. ($W_{\text{GPL}}=0.5\%,L/h=30,\left(k_{1},k_{2}\right)=\left(0.01,0.001\right)$).

$w_{c}$	Present	TSPM	**Ref.** [18]
0	86.4108	86.4269	86.4108
0.2	88.2526	88.2655	88.2526
0.4	93.7780	93.7811	93.7780
0.6	102.9867	102.9738	102.9867
0.8	115.8788	115.8435	115.8788
1.0	132.4540	132.3903	132.4540

### 5.2 Thermal buckling

Table 6 shows that the critical buckling temperature rise declines significantly with increasing length to slenderness ratio and increases with increasing base stiffness, where X-GPLRC beams have a higher critical buckling temperature, so that beams with more GPL content closer to the surface have greater bending stiffness and therefore more thermal buckling resistance.

**Table 6:** Dimensionless critical flexural temperature rise with different length to slenderness ratios and distribution patterns. (H–H, $W_{\text{GPL}}=0.3\%$).

$\left(k_{1},k_{2}\right)$	L/h	X-GPLRC	U-GPLRC	O-GPLRC	A-GPLRC
$\left(0,0\right)$	10	164.4260	133.5148	102.3058	123.0134
	15	74.5575	60.3122	46.0376	55.4964
	20	42.2378	34.1213	26.0099	31.3822
$\left(0.1,0\right)$	10	249.3593	218.4061	187.2391	207.9480
	15	159.4908	145.2035	130.9709	140.4310
	20	127.1711	119.0126	110.9432	116.3168
$\left(0.1,0.01\right)$	10	333.1851	302.1905	271.0649	291.7751
	15	243.3166	228.9879	214.7967	224.2581
	20	210.9969	202.7970	194.7690	200.1439

**Fig. 3:**
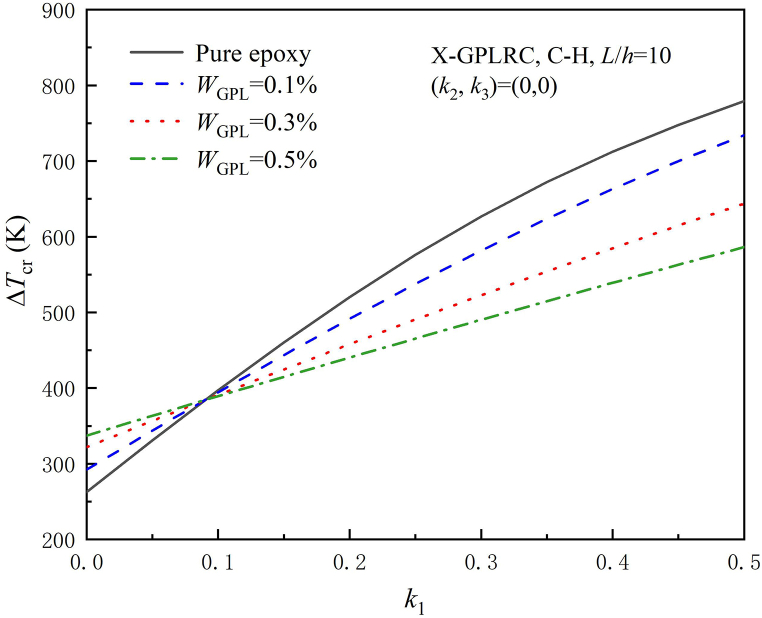
Effect of Winkler elastic stiffness coefficient on critical buckling temperature rise under different GPL weight fractions.

With different foundation stiffnesses and boundary conditions, Table 7 shows $\Delta T_{\mathrm{cr}}$ for X-GPLRC beams. Pure epoxy indicates a value with zero GPL weight fraction. For without elastic foundation, $\Delta T_{\mathrm{cr}}$ increases with the increase of GPL weight fraction, because more GPL dispersed will increase the bending stiffness of the beams. The opposite is true when placed on elastic foundation. Fig. 3 demonstrates that when the stiffness of Winkler elastic foundation is very small, $\Delta T_{\mathrm{cr}}$ grows as the GPL weight fraction rises. Therefore, the stiffness of the foundation determines how much the GPL weight fraction affects the thermal buckling of the GPLRC beam.

**Table 7:** Influence of GPL weight fraction on critical buckling temperature rise for various boundary conditions and elastic foundation stiffness. (X-GPLRC, L/h=10).

	$\left(k_{1},k_{2}\right)$	Pure epoxy	$W_{\text{GPL}}=0.1\%$	$W_{\text{GPL}}=0.3\%$	$W_{\text{GPL}}=0.5\%$
C–C	(0,0)	489.7248	542.0062	594.0576	620.6315
	(0.1,0)	613.3680	635.6128	657.0842	668.1951
	(0.1,0.01)	780.0347	760.8881	740.9100	731.2749
C–H	(0,0)	262.7601	292.3217	322.0220	337.2617
	(0.1,0)	397.0447	394.3730	390.9035	389.2898
	(0.1,0.01)	563.7114	519.6483	474.7293	452.3695
H–H	(0,0)	133.0972	148.6688	164.4260	172.5450
	(0.1,0)	301.9658	275.5992	249.3593	236.4582
	(0.1,0.01)	468.6325	400.8745	333.1851	299.5379

Next, the relationship between $\Delta T_{\mathrm{cr}}$ and $P\;/P_{\text{cr}}$ is analyzed for various GPL weight fractions, elastic foundation patterns, boundary conditions, and distribution respectively. $P_{\text{cr}}$ represents the critical buckling load. $P\;/P_{\text{cr}}<0$ indicates the axial tension force, $P\;/P_{\text{cr}}=0$ is the case when no axial force is applied, and $P\;/P_{\text{cr}}>0$ indicates the axial compression force.

**Fig. 4:**
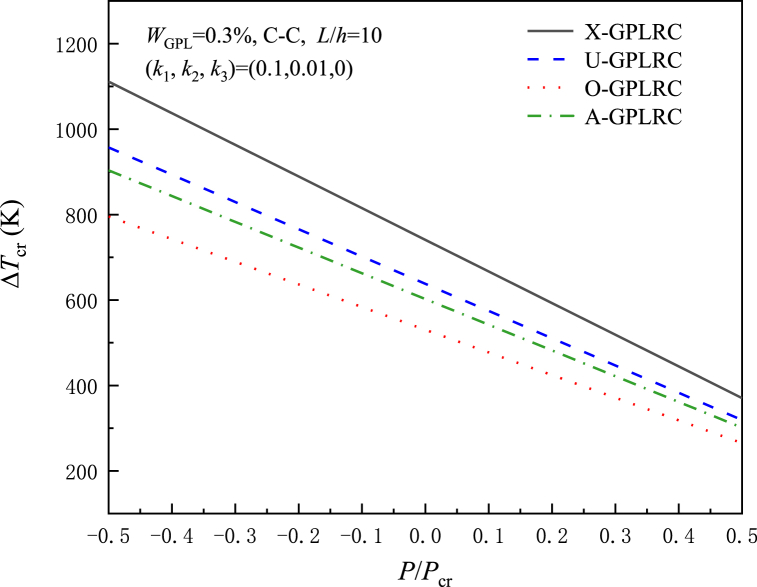
For different GPL distribution patterns, the impact of normalized axial forces on critical flexural temperature rise.

Fig. 4 shows the effect of different distribution modes with axial force $P\;/P_{\text{cr}}$ upon the critical buckling temperature rise $\Delta T_{\mathrm{cr}}$. The application of axial tension increases, as the axial compression force increases, $\Delta T_{\mathrm{cr}}$ decreases because the compression force creates an initial state of compressive stress in the beam, thus decreasing its stiffness. In addition, X-GPLRC beams exhibit the highest variation in critical buckling temperature, while the lowest variation is in O-GPLRC beams, which is because the more GPL content near the surface location, the better the bending stiffness of the beam.

**Fig. 5:**
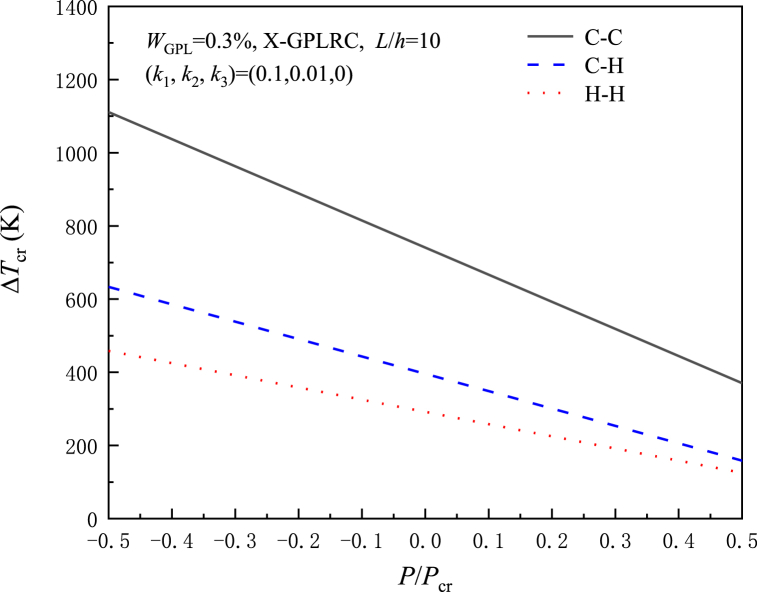
Effect of normalized axial forces on the critical buckling temperature rise of X-GPLRC beams under different boundary conditions.

Fig. 5 shows the relationship with the axial force $P\;/P_{\text{cr}}$ for various boundary conditions. In this case, as the axial compression force increases, $\Delta T_{\mathrm{cr}}$ is greatest for the C–C beam, then the C–H beam and finally the H–H beam.

**Fig. 6:**
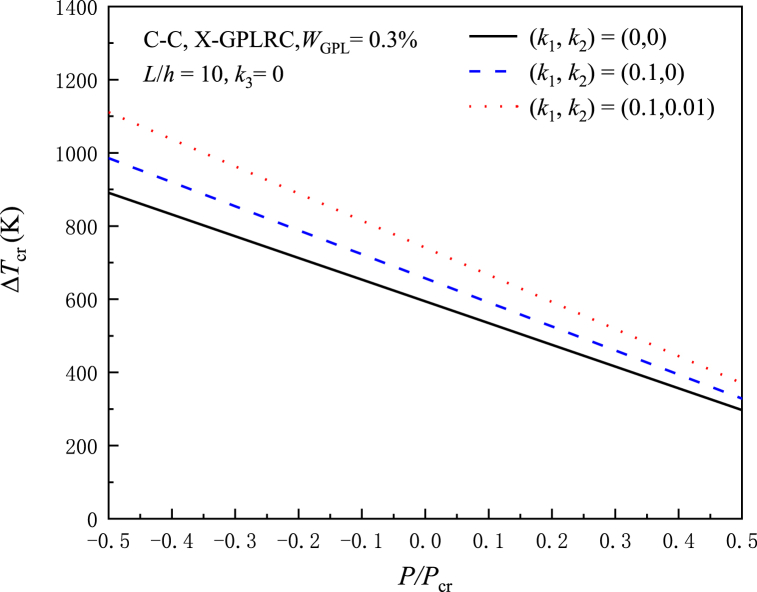
At different elastic stiffnesses, the critical buckling temperature rise of X-GPLRC beams is influenced by normalized axial forces.

The effect of different elastic stiffnesses with normalized surface internal forces $P\;/P_{\text{cr}}$ on $\Delta T_{\mathrm{cr}}$ is shown in Fig. 6. $\left(k_{1},k_{2}\right)=\left(0,0\right)$ for on elastic foundations, $\left(k_{1},k_{2}\right)=\left(0.1,0\right)$ for linear Winkler elastic foundations and $\left(k_{1},k_{2}\right)=\left(0.1,0.01\right)$ for Passant elastic foundations. Shear layer stiffness $k_{2}$ has a more noticeable influence than Winkler linear foundation stiffness $k_{1}$ when $\Delta T_{\mathrm{cr}}$ increases with increasing foundation stiffness. This can also be explained by the expression Eq. (53) obtained by TSPM. The increase of $k_{1}$ and $k_{2}$ leads to the increase of $\Delta T_{\mathrm{cr}}$. The dimensionless Eq. (52) and critical buckling temperature rise $\lambda_{1}$ of C–C beams can be expressed as follows:

(53) $$\begin{array}{l} a_{55}=\frac{\kappa A_{55}}{A_{110}},a_{11}=\frac{A_{11}}{A_{110}},c_{11}=\frac{C_{11}}{A_{110}},e_{11}=\frac{{B_{11}}^{2}\pi^{2}}{A_{11}A_{110}L^{2}}\text{,}\\ d_{11}=\frac{D_{11}\pi^{2}}{A_{110}L^{2}},b_{11}=\frac{B_{11}}{A_{110}L},k_{1}=\frac{K_{1}L^{2}}{\pi^{2}A_{110}},k_{2}=\frac{K_{2}}{A_{110}},k_{3}=\frac{K_{3}L^{4}}{\pi^{2}A_{110}} \end{array}$$

(54) $$\lambda_{1}=\frac{4\left(d_{11}-e_{11}\right)\left(3k_{1}+4k_{2}\right)+a_{55}\left\{3k_{1}+4\left[4\left(d_{11}-e_{11}\right)+k_{2}\right]\right\}}{4c_{11}\left[a_{55}+4\left(d_{11}-e_{11}\right)\right]}$$

**Fig. 7:**
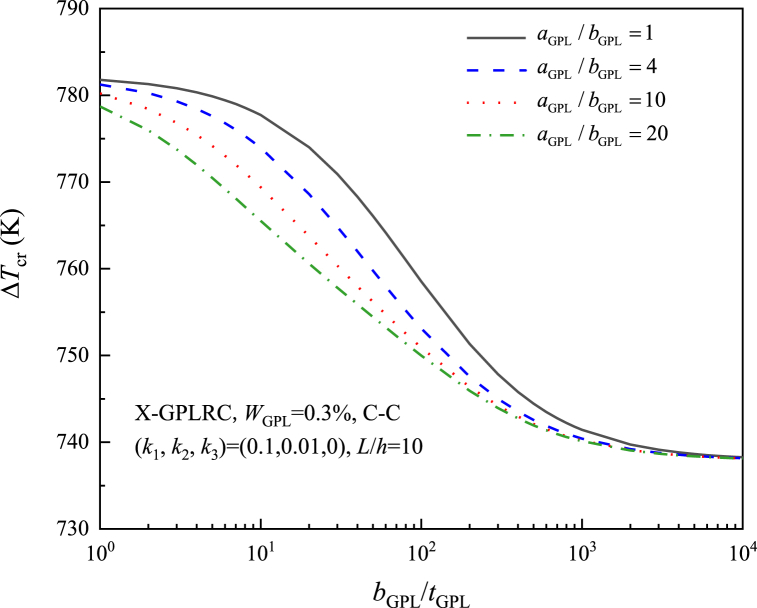
Effect of GPL geometric shape on the critical buckling temperature rise of X-GPLRC beam on elastic foundation.

Fig. 7 illustrates the influence of the GPL geometry on the $\Delta T_{\mathrm{cr}}$ of the X-GPLRC beam for a foundation stiffness of $\left(k_{1},k_{2}\right)=\left(0.1,0.01\right)$. With a constant width $b_{\text{GPL}}$, a larger $a_{\text{GPL}}/b_{\text{GPL}}$ indicates a larger GPL surface area, while a larger $b_{\text{GPL}}/t_{\text{GPL}}$ means less monolayer GPLs are contained. As shown in the figure, the $\Delta T_{\mathrm{cr}}$ decreases with increasing the GPL width-to-thickness ratio or aspect ratio, whereas it tends to be constant when $b_{\text{GPL}}/t_{\text{GPL}}>10^{3}$, which is contrary to this case without elastic foundations studied in Ref. [16] for GPLRC plates. As a result, the foundation stiffness determines how the GPL geometry affects the thermal buckling of GPLRC beams.

### 5.3 Thermal postbuckling

Following is a study of the analysis of thermal postbuckling of beams supported by nonlinear elastic foundation. First, the relationship between postbuckling temperature change ΔT and deflection of X-GPLRC beams at different slenderness ratios under H–H boundary is given, $w_{c}$ is the dimensionless central deflection. Table 8 demonstrates that the postbuckling temperature reduces noticeably as the slenderness ratio rises.

**Table 8:** Thermal postbuckling temperature versus deflection of X-GPLRC beams at different length to slenderness ratios at the H–H boundary ($W_{\text{GPL}}=0.3\%,\left(k_{1},k_{2},k_{3}\right)=\left(0.1,0.01,0.1\right)$).

$w_{c}$	L/h=10		L/h=15		L/h=20	
	Present	TSPM	Present	TSPM	Present	TSPM
0.0	333.1851	333.1851	243.3166	243.3166	210.9969	210.9969
0.1	337.9496	337.9496	245.7886	245.7881	212.6663	212.6660
0.2	352.2456	352.2456	253.2055	253.2055	217.6765	217.6765
0.3	376.0811	376.0816	265.5769	265.5772	226.0384	226.0385
0.4	409.4717	409.4719	282.9175	282.9175	237.7699	237.7699
0.5	452.4369	452.4366	305.2480	305.2478	252.8980	252.8979
0.6	505.0029	505.0026	332.5974	332.5970	271.4622	271.4619
0.7	567.2053	567.2040	365.0039	365.0034	293.5178	293.5174
0.8	639.0872	639.0830	402.5182	402.5165	319.1453	319.1436
0.9	720.6900	720.6907	445.2057	445.2004	348.4534	348.4540
1.0	812.0862	812.0888	493.1361	493.1374	381.6150	381.6157

**Fig. 8:**
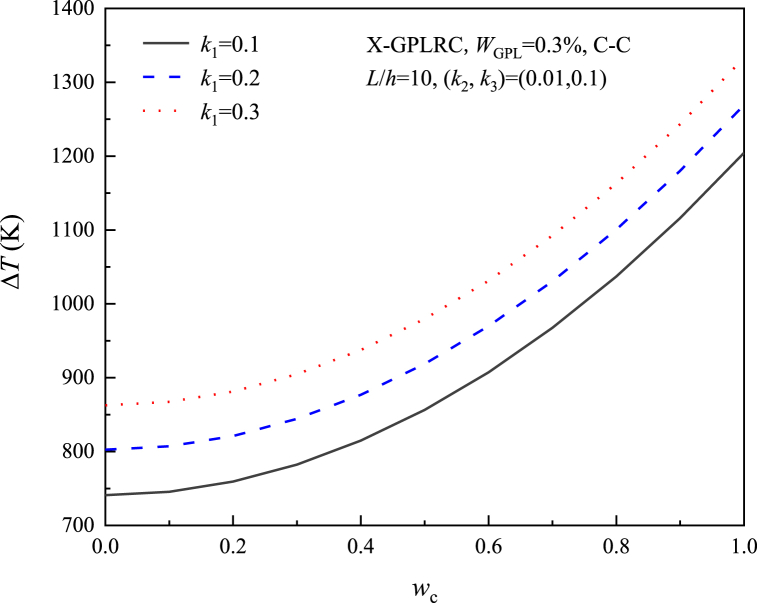
Effect of linear Winkler foundation on the thermal postbuckling path of X-GPLRC beams.

**Fig. 9:**
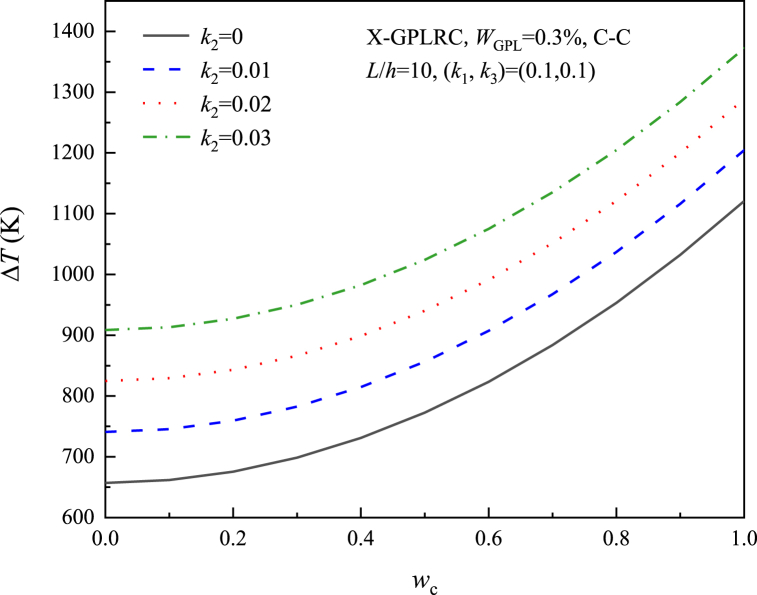
Effect of Pasternak foundation on the thermal postbuckling path of X-GPLRC beams.

**Fig. 10:**
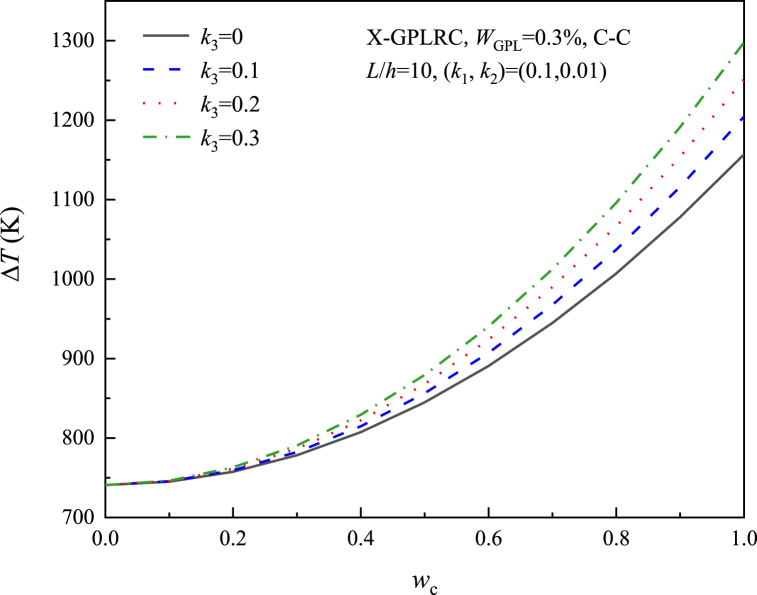
Effect of nonlinear Winkler foundation on the thermal postbuckling path of X-GPLRC beams.

**Fig. 11:**
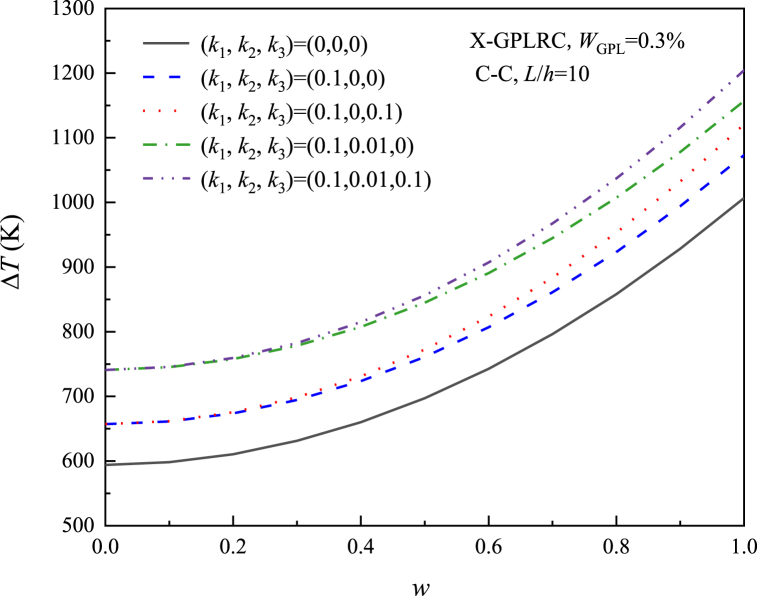
Effect of different elastic stiffness on the thermal postbuckling path of X-GPLRC beams.

Fig. 8, Fig. 9, Fig. 10, Fig. 11 study the relationship between the three elastic foundation coefficients and the postbuckling temperature change ΔT. From the figures, it can be obtained that the ΔT of the X-GPLRC beam increases as the elastic foundation coefficient increases, while the thermal postbuckling path changes when placed on a nonlinear Winkler foundation. This phenomenon can also be explained by Eq. (54) obtained by TSPM, by substituting Eqs. (55), (56) into Eq. (54), the thermal postbuckling equilibrium path is obtained, where $W_{m}=\eta w_{c}$, with the nonlinear stiffness coefficient $k_{3}$ appearing only in $\lambda_{2},A_{30}$. Therefore, as $k_{1},k_{2},k_{3}$ increases, the postbuckling temperature change increases. $k_{3}$ does not affect the postbuckling temperature change when $w_{c}=0$.

(55) $$\lambda_{T}=\lambda_{1}+\lambda_{2}{\left(W_{m}/2\right)}^{2}-2A_{30}\lambda_{2}{\left(W_{m}/4\right)}^{4}+\cdots$$

where

(56) $$A_{30}=\frac{\left[a_{55}+36\left(d_{11}-e_{11}\right)\right]\left(a_{55}+4d_{11}-4e_{11}\right)k_{3}}{8\left\{520a_{55}\left(d_{11}-e_{11}\right)k_{1}+1872{\left(d_{11}-e_{11}\right)}^{2}k_{1}+{a_{55}}^{2}\left[576\left(-d_{11}+e_{11}\right)+13k_{1}\right]\right\}}$$

(57) $$\begin{array}{l} \lambda_{2}=\{\{(27648{a_{55}}^{4}{\left(d_{11}-e_{11}\right)}^{2}\left(35k_{3}+16a_{11}\pi^{2}\right)+\\ \left[{a_{55}}^{2}+52a_{55}\left(d_{11}-e_{11}\right)+576{\left(d_{11}-e_{11}\right)}^{2}\right]{\left(a_{55}+4d_{11}-4e_{11}\right)}^{2}{k_{1}}^{2}\left(1215k_{3}+572a_{11}\pi^{2}\right)-\\ 48{a_{55}}^{2}\left(a_{55}+4d_{11}-4e_{11}\right)\left(d_{11}-e_{11}\right)k_{1}\left[48\left(d_{11}-e_{11}\right)\left(715k_{3}+332a_{11}\pi^{2}\right)+a_{55}\left(1575k_{3}+736a_{11}\pi^{2}\right)\right]\}\}/\\ \{4c_{11}\{110592{a_{55}}^{4}{\left(d_{11}-e_{11}\right)}^{2}+384{a_{55}}^{2}\left[-23{a_{55}}^{2}-590a_{55}\left(d_{11}-e11\right)-1992{\left(d_{11}-e_{11}\right)}^{2}\right]\left(d_{11}-e_{11}\right)k_{1}+\\ 143\left[{a_{55}}^{2}+52a_{55}\left(d_{11}-e_{11}\right)+576{\left(d_{11}-e_{11}\right)}^{2}\right]{\left(a_{55}+4d_{11}-4e_{11}\right)}^{2}{k_{1}}^{2}\}\} \end{array}$$

**Fig. 12:**
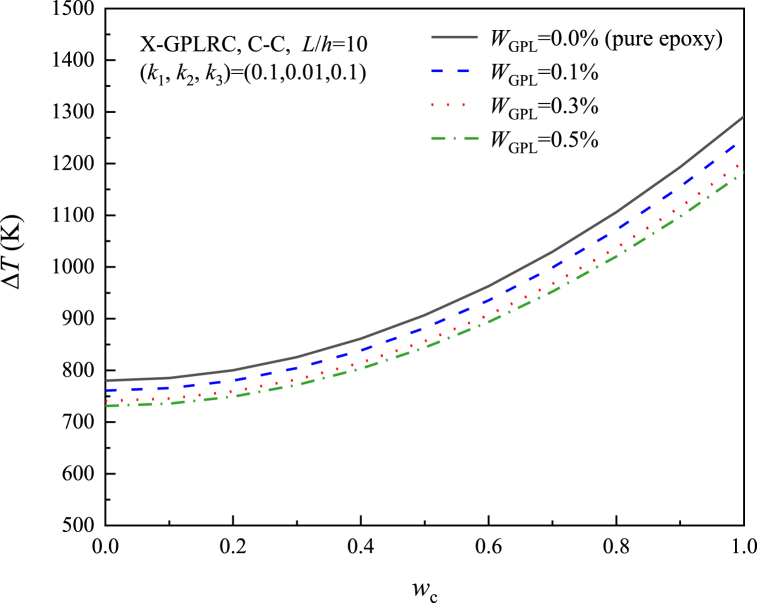
Effect of GPL weight fraction on the nonlinear foundation upon the thermal postbuckling path of X-GPLRC beams.

**Fig. 13:**
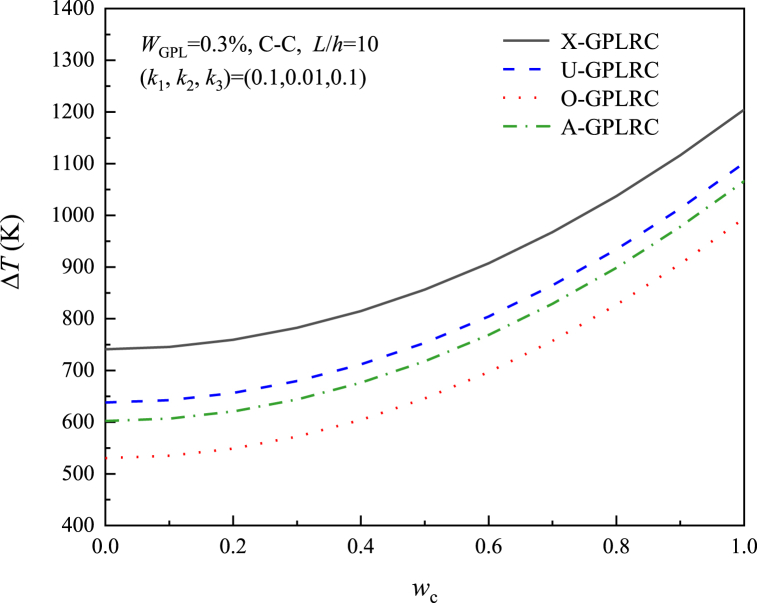
Effect of GPL distribution mode on thermal postbuckling path of beams on nonlinear foundations.

Fig. 12 depicts the thermal postbuckling equilibrium paths of X-GPLRC beams placed on nonlinear elastic foundations for different GPL weight fractions, where the postbuckling temperature variation decreases with increasing GPL content, in contrast to the results without elastic foundations. As a result, the foundation stiffness determines how the GPL weight fraction affects the thermal postbuckling of GPLRC beams. The effect of the thermal postbuckling equilibrium path of the GPL distribution pattern is shown in Fig. 13. X-GPLRC beams can withstand higher temperatures in the postbuckling phase, next to U-GPLRC and A-GPLRC beams, while O-GPLRC beams are subjected to the lowest temperatures due to the low GPL content near the top and bottom locations.

**Fig. 14:**
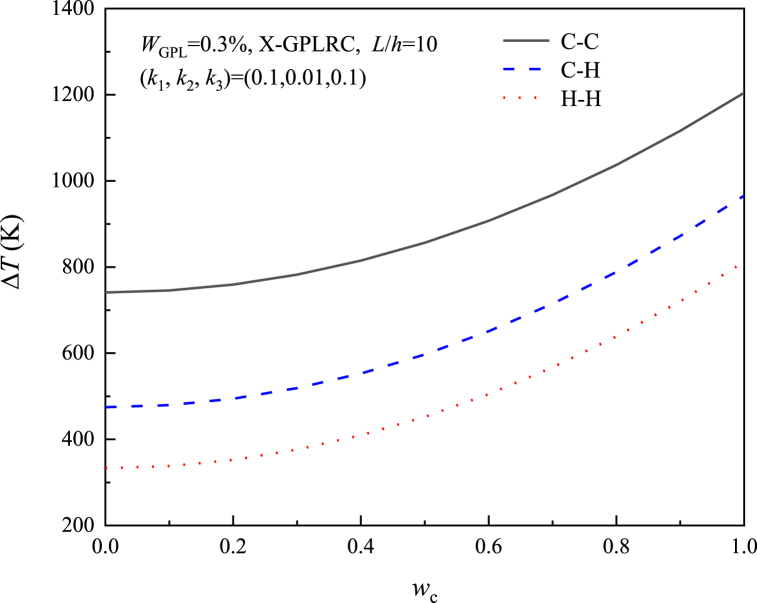
Effect of boundary conditions on nonlinear foundations on the thermal postbuckling path of beams.

Fig. 14 depicts the impact of various boundary conditions on ΔT for the X-GPLRC beam placed on a nonlinear elastic foundation. It is clearly obtained that the X-GPLRC beam has the maximum ΔT with C–C boundary conditions, while the H–H beam has the lowest ΔT.

**Fig. 15:**
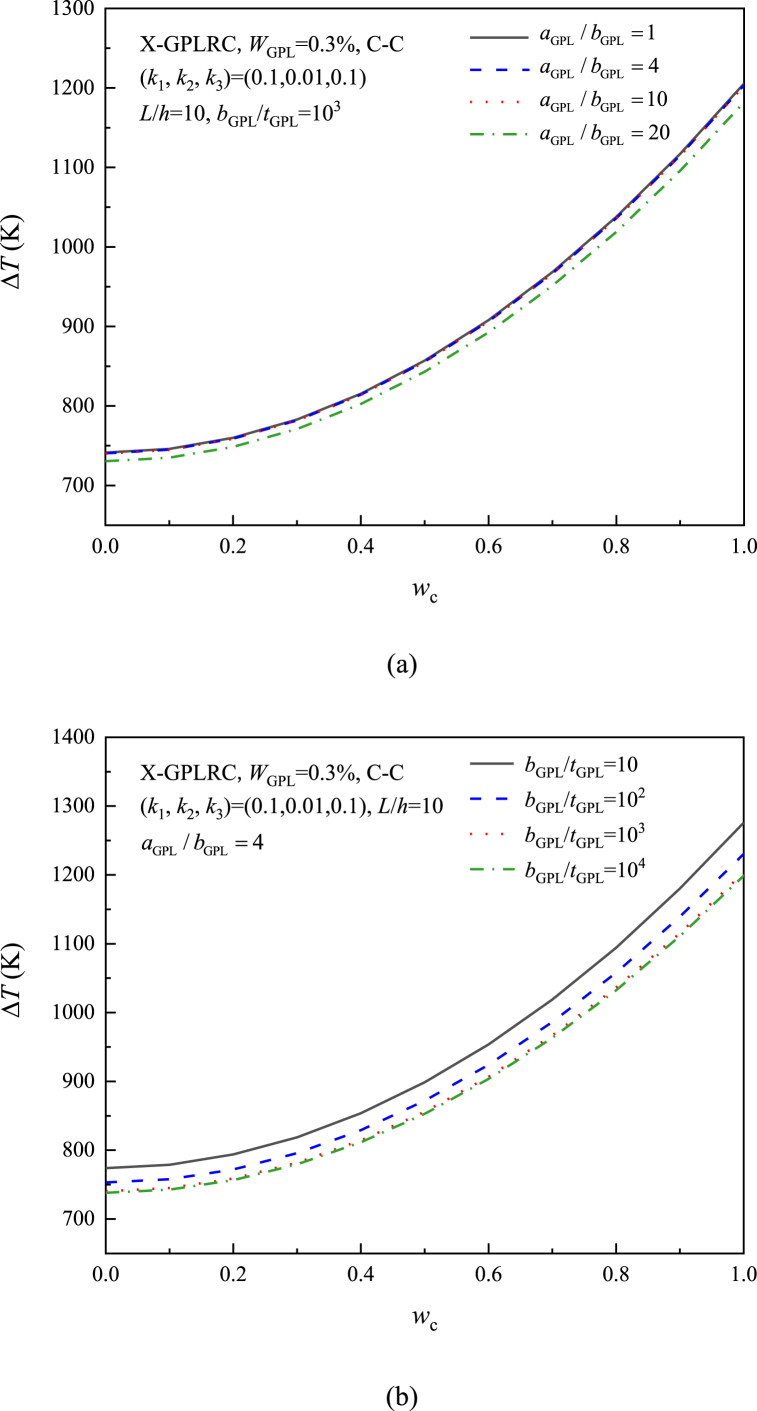
Effect of GPL geometry on nonlinear foundations on the thermal postbuckling path of X-GPLRC beams: (a) aspect ratio; (b) width-to-thickness ratio.

Fig. 15 (a) and (b) study the impact of GPL width-to-thickness ratio and aspect ratio on ΔT. The outcomes show that the thermal postbuckling temperature variation is lower for beams with larger aspect ratios or width-to-thickness ratio, in contrast to the case of inelastic foundations. As a result, the foundation stiffness determines how the GPL geometry affects the thermal postbuckling of GPLRC beams. It should be noted that the effect of GPL is considered more important than the effect of aspect ratio, but this effect is almost constant when the width-to-thickness ratio $b_{\text{GPL}}/t_{\text{GPL}}>10^{3}$.

**Fig. 16:**
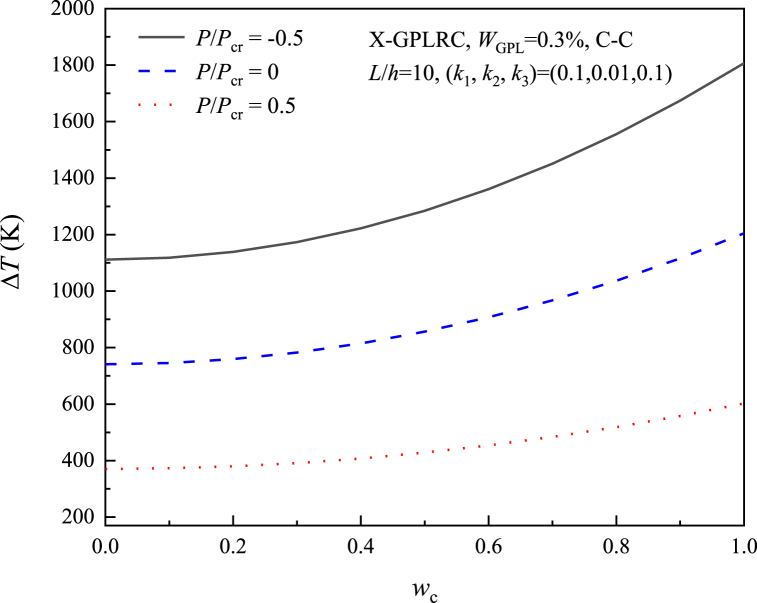
Effect of axial force on the postbuckling path of X-GPLRC beam on the nonlinear foundation.

Fig. 16 shows the application of different normalized axial forces $P/P_{\text{cr}}\left(P/P_{\text{cr}}=-0.5,0,0.5\right)$, it can be seen that the application of tensile force can lead to a higher change in the postbuckling temperature, the higher the deflection the faster the postbuckling temperature increases, but the application of compressive force leads to a lower thermal postbuckling curve, due to the creation of an initial compressive stress state that reduces the stiffness of the beam.

## 6 Conclusions

In this paper, the functionally graded multilayered GPLRC beams on nonlinear elastic foundations with the combined effect of homogeneous temperature rise and axial force are obtained by DQM for the critical buckling temperature rise and thermal postbuckling balance paths. Calculated results show that.

1. Decreasing the axial tension force and slenderness ratio can increase the critical buckling temperature.
2. Linear Winkler foundations, Passant foundations, and nonlinear Winkler foundations can reinforce thermal postbuckling performance, while thermal postbuckling paths are different from others when placed on nonlinear Winkler foundations.
3. The effects of GPL geometry and weight fraction on the thermal buckling and postbuckling of functionally graded multilayered GPLRC beams depend on the foundation stiffness.
4. GPL has the best performance in thermal buckling and postbuckling according to the distribution pattern of X-GPLRC, so more GPL should be added near the top and bottom when designing graphene functionally graded materials.

## Author contribution statement

Ying Lv: Conceived and designed the experiments; Performed the experiments; Analyzed and interpreted the data; Contributed reagents, materials, analysis tools or data; Wrote the paper. Jing Zhang: Conceived and designed the experiments; Performed the experiments. Lianhe Li: Conceived and designed the experiments; Contributed reagents, materials, analysis tools or data.

## Data availability statement

No data was used for the research described in the article.

## Declaration of competing interest

The authors declare that they have no known competing financial interests or personal relationships that could have appeared to influence the work reported in this paper.
